# “More” work for nurses: the ironies of eHealth

**DOI:** 10.1186/s12913-023-09418-3

**Published:** 2023-04-27

**Authors:** Susanne Frennert, Lena Petersson, Gudbjörg Erlingsdottir

**Affiliations:** 1 Department of Design Science, Lund University, Lund, Sweden; 2 Department of Health and Welfare, Halmstad University, Halmstad, Sweden

**Keywords:** eHealth applications, Invisible work, Articulated work, Nurses, Digital health

## Abstract

**Background:**

eHealth applications are considered a technological fix that can potentially address some of the grand challenges in healthcare, including burnout among healthcare professionals, the growing burden of patients with chronic conditions, and retaining and recruiting healthcare professionals. However, as the deployment of eHealth applications in healthcare is relatively novel, there is a lack of research on how they affect the work environment of healthcare professionals. This study explores how work evolves—particularly for nurses—during the utilisation of three eHealth applications.

**Methods:**

The study is a qualitative case study with an interpretive approach. The utilisation of three different eHealth applications was studied. Seventy-five healthcare professionals were interviewed, most of whom were nurses (*n* = 47). Interviews were transcribed verbatim and qualitative content analysis was used to analyse the text.

**Results:**

Three main themes were identified: *work that is ignored and overlooked; actions needed to complete visible work*; and *more sedentary work activities*. The findings suggest that work surrounding the utilisation of eHealth applications in care practices is mostly performed by nurses. While the promise of more efficient workflows resulting from healthcare’s digital transformation may be realised to different degrees, the utilisation of eHealth applications creates additional invisible labour for nurses.

**Conclusion:**

We identified through our analysis that the extra work created by eHealth applications is invisible at the organisational level. Most of the invisible labour was performed by nurses, who were engaged in utilising the eHealth applications. This needs to be recognised when implementing eHealth applications in care practices.

## Introduction

eHealth as a development in healthcare has been prominent for more than two decades since Eysenbach’s oft-cited article [1]. Much of the work within the area aims to develop and deploy eHealth applications to digitally support patient care [2]. Dominant discourse on eHealth applications promises that they will improve efficiency in healthcare provision and increase accessibility for patients [3]. However, there are still uncertainties regarding the impact of eHealth applications on health outcomes and healthcare work [4–6]. Regarding health outcomes for patients, there are studies indicating that remote data sharing and self-monitoring can reduce hospital readmission [7], medication errors [7], and hospitalisation [8]. These moderate findings suggest eHealth applications may have a positive impact on health outcomes. While there are reports on eHealth applications supporting healthcare workers, there are also indications that they may change and even increase the workload for healthcare professionals, especially for nurses. A study on patient-accessible electronic health records (PAEHRs), found evidence for increased workloads for nurses [9]. Another study, on Swedish primary healthcare nurses’ perceptions of using eHealth in support of patient management, found similar findings [10], as nurses claimed to be in the midst of digital chaos. These findings are troublesome, as nurses are the largest professional group in the healthcare system and many of them are debating leaving the profession due to feeling overworked [11]. Notwithstanding the extra work eHealth applications seem to impose on nurses, the Swedish government, and other nations—i.e. Australia, the UK, and Denmark [12]—have provided billions of dollars to support the digital transformation of their healthcare systems and the increasing use of eHealth applications [13]. Given these growing investments in eHealth, we argue that there is a need to understand how and why the implementation and use of eHealth applications in healthcare may have problematic consequences for nurses.

The aim of the paper is to raise awareness on the impact of eHealth applications on nurses’ work, informing decision makers, eHealth developers, and managers and helping them move toward a future working environment for nurses where there are fewer negative consequences related to eHealth deployment. The paper starts with providing a theoretical foundation of invisible and articulate work and offer a brief overview on the history of the nursing–technology relationship. It then presents case studies of three different eHealth applications in Swedish healthcare and highlight the impact the eHealth applications have on nurses’ work. Finally, the paper discusses the implications of our findings for nursing practice. We believe the observations and reflections presented in this paper are relevant to other contexts where eHealth applications are deployed.

### Theoretical frameworkInvisible and articulation work

The concept of ‘invisible work’ originated in the 1980s with Daniels study of women’s domestic work, referring to work that is unpaid and undervalued [14]. The concept has since expanded to include work that is ignored, marginalised, overlooked, underregulated, legally unprotected, or a mixture of all of the above [15]. Emotional labour, such as the engagement and detachment required in nursing, is an example of invisible work that is essential but often devalued [16, 17], as it is considered ‘natural’ and not an expertise or a competence [18]. Articulation work, which refers to the coordination and integration needed to complete visible work [19]. The boundaries between visible and invisible work or formal and informal work are fluid, and articulation work is carried out in the intersection between them [20]. For example, Oudshoorn (2008) explores the uptake of cardiac telemonitoring technology [21]. The nurses she studied were assigned a formal task, such as instructing patients on how to use the technology, attaching sensors, and sending ECG data. However, they also needed to spend time with patients to assure them that they could trust the technology. Thus, this invisible work of nurses in comforting and convincing patients to use the technology was pivotal for turning non-users of cardiac telemonitoring technology into users [21]. Similarly, Bødker and Juul Nielsen (2015) investigated online rehabilitation and the work required by patients and healthcare professionals [22]. They suggest that although telecare promises to deliver more efficient healthcare services and improves quality of care for patients, new invisible work practices emerge. These invisible work practices included patient recruitment and coordination, and integration of the telerehabilitation equipment into both the patients’ and the nurses’ everyday lives. Without this articulation work, the telerehabilitation infrastructure would not hold up, but at the same time this kind of work was devalued and unrecognised [22].

### The history of nursing–technology relationship

Nurses make up the largest segment of the healthcare work force globally and play a key role in any healthcare system [23]. Nursing is dominated by women and is considered a female-dominated service profession [24]. Thus, there is a strong gender bias toward females in nursing. Nursing is often associated with care and caring [25, 26]. The public discourse around nursing and care often implies ‘Nightingale stereotypes’ and Victorian ideals of women being empathic, sensitive, kind, compassionate, and self-sacrificing [26–28]. In contrast, technology is often linked to cultural values associated with stereotypical ‘male’ characteristics such as decision-making, logic, and rational thinking [29]. However, in environments where work involving technology is female-coded, it is often considered repetitive, mechanical, and the workers involved in its deployment are often depicted as low skilled, low-wage labourers [30, 31].

The use of technology in nursing has prevalent for centuries—e.g., thermometers, stethoscopes, monitoring machines, and computers [32], but the skills to incorporate technology into care practices are seldom acknowledged [31]. This may be due to the female-dominated nature of nursing and the perception of caring as a natural and inherited skill [26, 33, 34]. Hughes et al. (1971) argue that as a profession decreases in status and prestige, its work becomes less visible [35]. In healthcare, nursing professionals collaborate closely with physicians. The physicians’ profession is associated with medical science—focused on illness and curing—while the nurses’ profession is related to caring, focussed on helping patients living with illness [28]. Physicians, in comparison to nurses, have higher status and medical science is regarded as more prestigious than nursing [28, 34]. Thus, healthcare is comprised of larger social structures through which structures of power and knowledge are reproduced, and healthcare thus produces diverse technology usage by placing different expectations on different professions. In thinking about invisible and articulated work and structures of power, we can focus on the implications the utilisation of eHealth applications has for nurses.

As an example, Dillard–Wright (2019) examined the apparatus of power/knowledge electronic health records impose on nurses [36]. She likened the electronic health record to a panopticon, in which nurses are watched and governed. In other words, everything nurses enter into the electronic health record can be seen by patients, physicians, and nurse colleagues. On the one hand, nurses are subjected to power, as their work is under surveillance. On the other hand, nurses can exercise power, as it is their narratives about patients that are noted in the electronic health record [36]. The latter (exercising power) is challenged by self-monitoring eHealth applications, in which the patient’s own words are documented. Dillard–Wright (2019) argues that the electronic health record dictates the focus and work activities of nurses due to its scripts. Thus, scripts define, shape and control nurses’ work. The concept of “technological scripts” was coined by Akrich in 1992 [37] and refers to how developers’ ideas about how a certain technology should be used are inscribed into the technology like a manuscript; i.e., do A then B, etc. According to Dillard–Wright the technological scripts of electronic health records force nurses into certain standardised behaviours, thus overpowering innovation, individual decisions, and critical thinking [36].

Common themes in the literature on the nursing–technology relationship include training and digital literacy [38–40], and it is argued that the resistance to and implementation failures of eHealth applications are due to healthcare professionals’ non-technical skills and attitudes. However, Ziebland, Hyde, and Powell (2021) argue that eHealth applications may have unintended consequences for care work and need to be studied more critically and in more nuance in context, instead of assuming resistance to technology is a result of the characteristics of nurses or other healthcare professionals [41]. Following this argument, our study explores how work evolves for nurses during the utilisation of eHealth applications.

## Methods

### Case studies on evolution of invisible work

Qualitative case research with an interpretive approach [42, 43] was used to generate an understanding of how work evolves during the utilisation of three eHealth applications. Qualitative interpretive case studies facilitate in-depth studies on emerging eHealth applications in their natural setting [44]. Although past research [10, 22, 36, 45, 46] indicates that eHealth applications change and increase the work of nurses, the understanding of the phenomena is still limited. The method of qualitative interpretive case studies enables us to describe how the study participants interpret the work they are doing when deploying eHealth applications.

The study consisted of three case studies (see Table 1 for a short overview and Table 2 for detailed case descriptions). The three cases were selected as they provide data across five dimensions: (1) different groups of patients (primary care patients, patients with haemodialysis, and the aftercare of patients with heart failure); (2) different kinds of healthcare professionals (primary care nurses, hospital nurses from two different medical specialities, physicians, psychologist, managers, and administrators); (3) different eHealth applications (an eHealth application for digital entry and contact to and with primary care, an eHealth application for intermediate remote monitoring, and an eHealth application for continuous remote monitoring); (4) different parts of the healthcare sector (primary care and two types of specialist care); but at the same time are part of (5) the same cultural context (Swedish healthcare).

**Table 1:** Overview of the cases

	**Case 1**	**Case 2**	**Case 3**
Group of patients	primary care patients	patients with haemodialysis	aftercare of patients with heart failure
Kinds of healthcare professionals	17 nurses 6 physicians 3 managers 2 administrators 2 psychologists 1 physiotherapist	21 nurses 8 physicians 1 administrator	9 nurses 3 physicians 2 managers
Gender	27 female 4 male	25 female 5 male	10 female 4 male
Kinds of eHealth applications	digital entry to primary care	continuous monitoring	intermediate monitoring
Parts of the healthcare sector	primary healthcare	specialist care	specialist care
Cultural context	Sweden

**Table 2:** Summaries of the three cases

Case 1: An eHealth application to support digital entry to primary care
In 2016, physcians developed an eHealth application to support digital entry to primary care, with the aim of providing healthcare professionals with the ability to triaging patients digitally, thereby increasing accessibility and improving care effiency. Tha application enables registered patients to contact their primary care centre digitally, answering questions about their health and reasons for contacting a care provider, which are then compiled into a medical report that are visible to healthcare centre employees. Patients request are first assessed by a nurse, who can ask follow up questions via chat or video and decide if the patient’s request can be handled by a nurse or if a physician or other healthcare professionals need to be involved. Physcians or other healthcare professionals can be invitaded to participate in online communication regarding the errend (the communication between healthcare professionals is not visible to patients), or the patient errend can be delegated to another healthcare professional via the eHealth application. The application can also be used to iniate contact with patients regarding medical results and appointments This case study focus on the deployment of the eHealth application at three primary healthcare centres, in total, 31 interviews were conducted (17 nurses, 6 physicians, 3 managers, 2 administrators, 2 psychologists and 1 physiotherapist)
Case 2: An eHealth application for continuous monitoring
In 2014, a professor in computer science and his colleagues developed an eHealth application for remote monitoring of patients experiencing kidney failure and who require home dialysis. The development of the application was based on the study of healthcare professionals work, mainly the work of nurses, and involved digitalising manual patient data reporting in close collaboration with healthcare representatives, including physcians and nurses. Patients are remotely monitored through a encrypted tablet, which enables secure login without identification. Each patient has their own unique care profile in the eHealth application and the healthcare professionals monitor the dialysis treatment at home, in real time, by automatically transmitting health parameters such as weight and blood pressure through Bluetooth technology. The application presents the results to healthcare professionals and patients through visualisations and trend curves. Patients either perform their own dialysis four times a day or have assisted treatments, in which municipal nurses perform the home dialysis and use the same tablet to report data as patients who perform their own treatment. Patients can communicate with healthcare professionals through video calls, chat messages, and photos if necessary. Nurses attend daily to the patient-reported data while the physicians mostly attend to the trend curves and patient data overview before physical visits (these normally take place every six weeks for each patient), when a prescription is changed for a patient, and/or when a patient’s values are unstable and require attention. Sometimes, the nurses also initiate a conversation with the responsible physician regarding medical data in the eHealth application that need to be addressed This case study focus on the deployment of the eHealth application at clinics in five different hospitals, in total of 30 interviews were conducted (21 nurses, 8 physicians, and 1 administrator)
Case 3: A digital eHealth application for intermediate monitoring
In early 2000s, a development company created a digital eHealth application aimed at making healthcare more efficient. The first version of the application was tested and further developed in collaboration with a clinic specialised in aftercare for patients following myocardial infarction. The eHealth application enables patients to exchange information and communicate with healthcare professionals via a web application. Patients log in using their individual ID and enter their predetrimened vital parameters in the downloadable webapplication for smartphones, tablets or computers. The application. Includes a chat function that allows communication between healthcare professionals and patients. Patients have a unique care profile in the eHealth application where they report health parameters like blood pressure and weight. Patients use their own technical equipment, and if a particular patient does not have a blood pressure cuff, for example, they do not report their blood pressure. The application creates a visualisation of the patients condition, and an assessment is shown to healthcare professionals using different colours: red indicating urgency, yellow indicating a need for attention, and green indicating that everything is well with the patient. At the three hospital clinics studied, patients did not have scheduled times for reporting data into the application. Some patients reported regularly, regardless of their state of health, while others only reported when their health was deteriorating. Nurses assessed the patient-generated on a daily basis and are responsible for keeping track of patients, while physcians use the application before patient contact This case study, focus on the deployment of the eHealth application at three hospital clinics, in total 14 interviews were conducted (9 nurses, 3 physicians, and 2 managers)

The companies behind the three eHealth applications provided contact information with healthcare practices using their applications. The researchers then contacted and gained access to the practises described in Table 2, where the three applications were in use. The researchers had no former relations with these healthcare practices.

Individual semi-structured interviews [47] were conducted by all three authors in order to capture the work nurses and other healthcare professionals perform to make the eHealth applications part of their everyday work. A purposeful sampling included 75 interviews with nurses (*n* = 47), physicians (*n* = 17), administrators (*n* = 3), psychologists (*n* = 2), a physiotherapist (*n* = 1) and managers (*n* = 5). The Interviewees were selected due to their experience working with one of the three eHealth applications i.e. we wanted to understand the meanings and interpretations of the perceived impact on the working environment the eHealth applications had among different healthcare professionals. The interviewees had a wide age range, from their 20 s to 60 s, with the majority being middle-aged. They also had varying levels of work experience in the healthcare sector, ranging from a few years to over three decades The semi-structured interviews followed an interview guide, lasted 30–90 min, and were recorded and transcribed verbatim. In the interview guide we asked questions such as: could you tell me about a situation in which you used the eHealth application? How did you experience that situation? Who is involved in using the eHealth application at your work place? Has the eHealth application had any impact on your working environment? If so, how? Has the eHealth application had any impact on your relationship with colleagues and other healthcare professionals? etc. Through these and other questions, we sought to gather comprehensive information about the experiences and perspectives of a wide variety of healthcare professionals regarding the eHealth applications. SF conducted the interviews in case 1, LP in case 2, LP and GE in case 3. In addition, field observations were carried out at primary care centres. The observations were planned walk-along sessions [48] and took place during the initial training sessions as well as during half- and full days when the nurses conducted their everyday care work, with a total of five full days and four half days observations. The observations were either conducted by SF or GE, focusing on the routines and habits among nurses when interacting with other healthcare professionals, patients, and various technologies. During the observations, fieldnotes were taken. While interview transcripts form the basis for our analysis, the analysis is strengthened and informed by field observations.

### Analysis

The approach used for our analysis can be broken down into three steps. First, each case was analysed separately through inductive qualitative content analysis [49] to ‘make meaning’ of each case. The identified themes of each case were compared and discussed within the research group. In the cross-case analysis we found interrelated paradoxes. The findings of this analysis have been published [50]. Three authors (SF, LP, and GE) discussed the findings of the inductive qualitative content analysis in the published paper and acknowledged that all three eHealth applications had more impact on nurses’ work than on the work of other healthcare professionals, as they perpetuated boundaries between professional groups due to ingrained power structures [50]. We found this interesting to explore further in this paper. Thus,we searched and read the literature on nursing–technology relationship and came across literature on invisible and articulate work. We used the insight from the literature on invisible and articulated work [14, 15, 18, 20] to conduct our deductive analysis of the data. In this second step, the empirical data for each case was read and reread several times through the lens of invisible and articulated work. We analysed the transcripts from all interviews (i.e., not only for nurses but also other healthcare professionals) and the fieldnotes by hand to get a deeper understanding of whether the eHealth applications had a different impact on nurses than the other healthcare professionals and if so, how?

After conducting separate analyses, we engaged in joint discussions and shared our interpretations of the empirical material. During this phase, we perceived that a common matter among the nurses was an increase in sedentary work resulting from the use of eHealth applications. Although this had not been previously described in the literature on invisible and articulated work, we recognized its significant and hidden impact on the nurses' working environment and health. We moved back and forth in the data and discussed our interpretations of the narratives portrayed in the transcripts [51]. All three authors have different backgrounds and our interpretations of the data were contrasted for similarities and differences [52], first within each case and then across the three cases [44]. An example of this process is the identification of sub-themes that was related to work that is expected to be carried out by nurses but is ignored or not valued by others. We identified that the eHealth applications added additional responsibility and increased complexity to nurses' work. The eHealth applications also required more supervision of data, which resulted in extra workload as they had to ensure the accuracy of both reported and unreported data. As we had very similar interpretations of the data, we iteratively constructed potential themes and sub-themes which we reviewed, defined and named [51]. The third step consisted of synthesising the findings [53].

## Results

A total of 75 healthcare professionals were interviewed, most of whom were nurses (*n* = 47). During data analysis we identified three main themes related to the work of nurses when utilising the three eHealth applications: *work that is ignored and overlooked, actions needed to complete visible work,* and *more sedentary work activities* (See Table 3). The interpretive qualitative analysis focused on invisible and articulated work, but we also found one theme that did not fit into the conceptual framework used in the analyses (the last theme). The two main themes consisted of six subthemes (see Table 3).

**Table 3:** Main themes and sub-themes

THEMES	SUB-THEMES
WORK THAT IS IGNORED AND OVERLOOKED	• Additional responsibility and increased complexity
	•Supervision of data
	• Reassurance about reported and unreported data
ACTIONS NEEDED TO COMPLETE VISIBLE WORK	•Interpretation of data
	•Layout/editing
	• Teaching patients to use and handle the devices
MORE SEDENTARY WORK ACTIVITIES	

### Work that is ignored and overlooked

Across all three eHealth applications, the idea that work that was conducted by nurses was to some extent ignored and overlooked by their organisations (i.e., work that was crucial but not highlighted by others; work that was rendered invisible by others) was identified in the data analysis. Invisible work was related to three sub-themes: (1) additional responsibility and increased complexity, (2) supervision of data, and (3) reassurance about reported and unreported data.

### Additional responsibility and complexity

In the cases of digital entry to primary care and intermediate monitoring, interviewees described that the eHealth applications did not replace pre-existing care pathways and care work. Instead, they added new work for nurses, running parallel to existing work. This was due to the fact that not all patients were able or willing to use the applications. As a result, nurses had to carry out both traditional and digital work routines, which added to their responsibilities and made their work more complex. This irony was explained by one of the nurses using the eHealth application for digital entry to primary care:

“it becomes more to keep in mind... as there are a lot patients phoning, we are stuck on the phone and then you get patients requests through the eHealth application and there you have...okay, you don't have to answer the whole request but you have to, within half an hour, give some kind of reply that you have seen the errand, so you have to get it up and running at the same time as there are a lot of calls and they want help fast…”

[Interviewee 64, nurse, using the eHealth application for digital entry to primary care]

As in the above quote, many nurses described how they combined their pre-existing routines with new digital care work routines.

However, in one of the primary healthcare centres using the eHealth application for digital entry, there were enough patients to allocate one or more nurses solely to digital work, while others had allocated time for pre-existing work routines. During these circumstances, nurses reported that the eHealth applications saved time as some of the digital work was done by patients; such as measuring their own health parameters at home and answering triaging questions online.

### Supervision of data

Pre-existing care work routines run on different logic than digital work routines. Pre-existing care work is characterised by synchronous communication with patients through phone or physical meetings and is typically scheduled one patient at a time. Digital work through eHealth applications involves asynchronous communication, allowing multiple interactions with different patients over time; i.e., patients use the eHealth applications when available and if time allows. Thus, nurses narrated their frequent management of the data generated by the eHealth applications in a similar manner as one of the nurses using the eHealth application for continues monitoring:

“I have to constantly check so that...or almost all the time, check if someone has sent something”

[Interviewee 4, nurse, using the eHealth application for continues monitoring]

Interviewees highlighted that the eHealth applications amplified data on patients’ state of health. This was described in most interviews as an added advantage. As one of the nurses said:

“We are more attentive. We can keep an eye on the patients who are at home. Vital parameters of patients are observed daily, and we can contact them if necessary…they don't have to wait for an appointment…we check on them constantly…it is positive.”

[Interviewee 41, nurse, using the eHealth application for intermediate monitoring]

At the same time, it became clear that it was the nurses who had to manage the increase in patient data. As explained by one of the physicians:

“The daily follow-up via the continues monitoring eHealth application, it is in the hands of the nurse, and they flag when there is something, when they feel ‘something is not right with the patient, and we need to think about it’ and so on”

[Interviewee 13, physician, using the eHealth application for continues monitoring]

The management of increased dataflow became an added but invisible burden for nurses, that required time and effort in an already fragmented workday.

### Reassurance about reported and unreported data

The added responsibility and complexity of digital work routines and data supervision also generated new working routines in terms of reassurance about reported and unreported data. The nurses described how they needed to contact patients via phone if the data from the eHealth applications was unclear or if the patient didn’t report data as expected. Often, nurses preferred to contact the patient through a phone call as they perceived it to be more efficient than a prolonged chat conversation. Judgements were that over the phone they receive direct answers and could instantly adapt and ask follow-up questions, while in the chat it could take time for the patient to answer; i.e., the conservation could be stalled due to slow response time on follow-up questions.

The eHealth applications for intermediate and continuous monitoring relied on daily patient-generated data. However, some patients reported irregularly. In such cases, nurses had to contact patients regarding unreported data when they didn’t receive any information. One nurse, using the eHealth application for intermediate monitoring mentioned the following:

“There are those who do not frequently report data…for example I had one patient who said when I called him and wondered why he didn't [he said] ‘I forgot to do that.’”

[Interviewee 32, nurse, using the eHealth application for intermediate monitoring]

Thus, nurses anticipated receiving patient data through the eHealth applications, and had to resort to workarounds when data wasn’t available. Workarounds were necessary due to patients’ (non)compliance with the logics of the eHealth applications.

### Actions needed to complete visible work

Our analysis suggested for visible care work to be completed, the nurses needed to (1) interpret data, (2) layout/edit data, and (3) teach patients how to use the applications. In this context, visible care work refers to physical check-ups, documentation in electronic health records, communication with other healthcare professionals, measurements, and standardised documentation.

### Interpretation of data

Alll three eHealth applications required patients to report their health status through measurments or descriptions. The design of the applications defined what measurements and numbers patients needed to report, and the nurses had to interpret the data to decide on appropriate actions. For example, in the eHealth application for digital entry to primary care, patients were asked to rate their pain level on a scale from 0–10 (0 for no pain and 10 for extreme pain). Consequently, nurses then needed to interpret what the ratings meant. Nurses described that many patients entered high numbers as they believed it would fast-track them to a physical medical appointment. One of the nurses, referring to the eHealth application for digital entry to primary care, complained:

“Levels of anxiety or pain are difficult to interpret through numbers…many people feel that I'm contacting you because I'm in a lot of pain, so of course I'm very much in pain, they rate it as ten…nuances disappears as it is just a number… it makes it hard to understand the severity and what actions are needed”

[Interviewee 60, nurse, using the eHealth application for digital entry to primary care]

Similarly, a nurse, referring to the eHealth application for intermediate monitoring, explained:

“The very structure of the program itself limits you. Because when you enter the application, many may have rated their health as bad...it can be red for all patients [red indicating urgency]. Just ‘oh, my God, everyone is very sick’…when you call them or set them up for a medical appointment, it turns out it is not an urgency. You receive a picture [of the patient’s health] that is perhaps worse than it really is"

[Interviewee 45, nurse, using the eHealth application for intermediate monitoring]

Nurses not only had to interpret patient-generated measuments and assess their urgency, but also to receive excessive and disorganised data, making it challenging to understand the overall picture. The standarised data collection through the eHealth applications meant that patients entered irrelevant data, requiring nurses to extract key information from large amounts of data.

### Layout/editing

In all cases, nurses were responsible for compiling eHealth application data and entering it into the patient’s electronic health records, which required significant lay out work and editing. The data did not transfer automatically, and nurses utilised different techniques to lay out and edit data. One nurse, using the eHealth application for digital entry to primary care, described her procedure as follows:

“You can copy/paste all the data, but it gets quite messy, I think anyway… I usually sort of summarise everything in the electronic health record, what I think is essential, symptoms and more”

[Interviewee 73, nurse, using the eHealth application for digital entry to primary care]

The nurse describes how she summarised the dialog and data from the eHealth application for digital entry to primary care while others copied and pasted most of the data generated in the eHealth application. Correspondingly, patient-generated data is portrayed differently in the electronic health record depending on who entered the data.

### Teaching patients to use and handle the applications

As the three different eHealth applications depend on patient-reported data, it is obviously important that patients use the applications. Different strategies were used to engage patients in the three different applications. For digital entry to primary care, no training was provided, while for continuous monitoring and intermediate monitoring, nurses were responsible for recruiting and training suitable patients. Nurses spent considerable time introducing the applications and training patients. When estimating the time spent on this task, a nurse, referring to the eHealth application for continues monitoring, explained:

“You see them [the patients] quite often at the start…you are even with them in their home, and sometimes you go to their home an extra time to make sure they are on top of things”

[Interviewee 24, nurse using eHealth application for continues monitoring]

Interviewees using the eHealth applications for continuous monitoring and intermediate monitoring highlighted that initial introduction and training were crucial when it came to utilising the eHealth applications. If patients failed to use the applications or failed to report data, the nurses would not be able to enter the measurements and standardised documentation and needed to contact patients by phone or chat. Thus, patient engagement was crucial in digital working routines.

In addition, patients contacted the nurses when they needed help with technical support for the eHealth applications for continuous monitoring and intermediate monitoring. At the initial introduction, patients received information about technical support from the application’s developers. However, when patients experienced problems with the technology, they contacted nurses for technical support. One nurse, who worked with the application for continuous monitoring, explained that their patients contacted them and asked for help when there are problems with the devices and that it is hard to say no to the patient. She exclaimed:

“You don't want to be technical support. That...that's not our job, but you get drawn into it”.

[Interviewee 15, nurse using eHealth application for continues monitoring]

### More sedentary work activities

While our analysis focused on invisible and articulated work, a consequence of increased digital work routines was decreased physical inactivity. Nurses complained about how the work in managing and supervising data rendered by the eHealth applications led to more sedentary work activities. They were able, and encouraged, to communicate with colleges through the applications, which resulted in less movement within the wards/primary care centres. Furthermore, sitting down in front of computer screens resulted in fatigue:

“You become tired considering that you have been sitting still for so long. At the same time, the time goes by very quickly when you are chatting with patients. The screen time, I think takes a lot of energy...you get more tired”

[Interviewee 58, using the eHealth application for digital entry to primary care]

The nurse describes how time passes quickly when chatting with patients. In a similar manner, many nurses described asynchronous communication with patients, particularly when working in the eHealth application for digital entry to primary care, as immersive. This in turn resulted in fewer regular breaks and more sedentary work.

## Discussion

This study aims to shed light on the less visible and obvious ways of how eHealth applications impact nurses’ work. The empirical data shows that these applications have increased workload for nurses by adding digital work routines to pre-existing ones. The irony is that the digital transformation of healthcare is often portrayed as new technology that is solving problems in new and more efficient ways [54], transforming organisations [55], and replacing pre-existing ways of working. However, the eHealth applications for intermediate monitoring and digital entry to primary care we studied did not replace pre-existing routines. Instead, nurses had to work in parallel practices amidst different logics. When confronting these differing demands, nurses are forced to resort to workarounds when utilising the eHealth applications. This kind of work is seldomly recognised and is therefore rendered invisible and undervalued. In the case of continuous monitoring, pre-existing routines were largely replaced by the eHealth application, but at the same time the nurses received more data demanding constant attention. Thus, the findings of the present study, as well as those of other studies [10, 19, 21, 46, 56–59], indicates that work that is made visible (e.g., triaging, measurements, documentation) is materialised in the eHealth applications while invisible work is not. Nevertheless, relational work with colleagues and patients was made possible through asynchronous communication via the chat-functionality of the eHealth applications, but the extent to which this relational work was required did not materialise when organising work schedules. As such, it went unacknowledged. Similar to the work of Lie et al. (2019), we found that relational work through asynchronous communication through written language was more demanding and time consuming than synchronous oral communication [60]. For example, asynchronous communication was perceived as more ineffective than synchronous communication through the phone when quick responses were needed from patients.

Our study found that the eHealth applications required nurses to take on the role of invisible data managers, keeping track of both reported and unreported data. Reported data must be interpreted, edited, and transferred into the electronic health record. It may seem like a straightforward process, but each step involves invisible labour that is often overlooked. Petient-generated data is often complex and fragmented, requiring nursing skills and knowledge of the patient to make sense of the data. Thus, patients’ interpretations of the objectives of the eHealth applications and their role as eHealth users differ. Accordingly, nurses need to align different data perspectives to make sense of the whole picture (e.g., the patient’s state of health).

Concerning articulated work, eHealth applications enter care work organisations with insufficient interoperability to pre-existing obligatory documentation systems; i.e., the electronic health record. As a result, nurses have to copy/paste, edit, and layout patient data to make it fit into obligatory documentation systems. Furthermore, to receive patient data, nurses must sometimes teach patients how to use and handle the eHealth applications for intermediate and continuous monitoring. When technical troubles arise, nurses become the first line of technical support, as patients reached out to nurses and not the company that developed the applications. Unreported data was also underlined as an issue in all three cases. In the eHealth application for digital entry to primary care, patients often failed to answer the system-generated questions and, as a result, the medical report to the nurses was incomplete, which was often solved by follow-up questions through the chat or via a phone call. In the case of intermediate and continuous monitoring, unreported data resulted in concerns about patients, which was often solved by phoning patients. As such, repair work (e.g., repairing communication) was needed to make the eHealth applications work in practice. This result is in line with the work of other scholars [21, 61, 62]. Based on the outcomes of this analysis, increases in digital work routines led to fewer physical activities and more sedentary work. Increased time spent in front of computer screens was perceived as tiring. According to the results of the study by Moreno–Llamas, sedentary behaviour and inactivity has a negative impact on an individual’s health [63].

What we did identify in the data in relation to digital entry to primary care, was that in one out of three primary care clinics, the number of patients using the digital entry was significant enough to divide nurses working exclusively on patients contacting the primary healthcare centre by phone or through the eHealth digital entry application. This division of duties reduced nurse’s perceived stress levels and made them feel more content with the eHealth application [64]. The nurses even expressed that they found it relaxing to work on indirect patient through asynchrounous conversations rather than direct patient contact through the phone. However, the nurses still had to move and summarise text between the eHealth application and the electronic health record, resulting in additional work.

In sum, nurses’ work, through the eHealth applications, became diverted from direct patient care toward data management. The findings indicate that eHealth applications require care, nourishment and supervision, otherwise they lose their utility. Their existence depends on patient-reported data. Thus, healthcare workers, particularly nurses, need to simultaneously take care of running the eHealth applications, encouraging patients to use the eHealth applications, and managing patient data. The care and nourishment of eHealth applications takes time and the invisible labour it requires seems to be directed toward nurses. This responsibility directed towards nurses may be explained by the fact that a physician’s profession is focused on illness and curing, while the nurses’ profession is related to caring, and is focused on helping patients living with illness [28]. eHealth applications for continuous and intermediate monitoring are developed to monitor patients’ everyday lives while living with illness and it may be seen as a ‘natural task’ for nurses to take care of the eHealth applications and the data they generate, as it keeps them up to date on patients’ vital parameters. Perhaps this nurturing of data, which goes largely unnoticed at the organisational level is the new form of caring, and the nurses assuming responsibility for nurturing this data is a way of caring for patients in a digital world. As the eHealth applications become incorporated into care practices, they increasingly influence caregiving and demands the nurses’ attention. This does not mean that eHealth has an overall negative effect; however, the extra work required in taking care of the eHealth applications and the data they generate becomes additional and invisible labour for nurses, which must be recognised by organisations, eHealth developers, and decision-makers as it has a profound effect on nurses’ work environment.

### Implications for practice

The present study has several implications for eHealth developers, managers, and decision-makers.

First, eHealth applications often rely on patient-generated data. Our empirical findings indicate that both reported and un-reported patient data are of concern to nurses. eHealth developers must make visible in the user interface for patients what is expected of them; namely, what kind of data to report and when.

Second, managers should critically examine their ways of framing work, acknowledging both visible and invisible work. They ought to question the status quo regarding what is perceived as/considered to be ‘work’ within the organisation. The point here is not to make informal and invisible work visible in digital eHealth applications, but to provide space for healthcare professionals to carry out invisible work [65]. That is, the ‘problem’ is the lack of a holistic organisation of work that comprises both visible and invisible work, pre-existing routines, and new digital routines. As eHealth applications become entrenched in the work of healthcare professionals, there needs to be an understanding that work routines change over time [66]. Norms and work routines are continuously being negotiated. The utilisation of eHealth applications at work is a dynamic process, which is not stable, and as such there needs to be room for flexibility in the scheduling of work hours.

Third, as eHealth applications, or at least the responsibility of utilising them in practice, seems to be directed towards nurses, decision-makers must question if this should be the case; is more work for nurses desirable? However, the idea might be to make nurses’ work easier and more efficient. Our empirical findings show that new digital work routines do not always replace pre-existing work routines. As a result, nurses need to work in parallel practices amidst different logics. Decision-makers need to have realistic expectations on the impact of eHealth applications in practice and raise questions about interoperability of different systems.

Fourth, our empirical findings indicate that increasing digital working routines leads to more sedentary work. In the long run, this may result in negative consequences for nurses’ health. To prevent the negative effects of sedentary work, managers need to support nurses in working with data rendered by eHealth applications with sit–stand desks and frequent ‘movement’ breaks. This recommendation may seem obvious, but our observations show that the working environment of nurses did not include the possibility of standing while doing desk work in all workplaces.

Fifth, it is important to use eHealth applications where they fit best and/or to make efforts to encourage patient use, as our data showed clear differences in how nurses perceived working with digital entry to primary care between different primary care clinics. Having enough patients using the eHealth application to justify dividing the work between in-house and digital workloads seems to be key for not having to continuously work in parallel practices and logics.

### Limitations

The current study is based on data from Sweden, which might affect its generalisability to other countries. However, as three eHealth applications were studied, the generalisability in a Swedish context is higher than if one only eHealth application had been examined. Additional international studies could validate the findings.

Because this study took place during the COVID-19 pandemic, interviews were performed either at a distance, using the Microsoft Teams application, or face-to-face, and this variation might be a limitation. However, according to [67], distance interviewing with videoconferencing services, such as Microsoft Teams, could be beneficial and even preferred. Another consequence of the COVID-19 pandemic that might constitute a limitation is that field observations only occurred in one of the cases due to restrictions.

The study is strengthened by the authors’ familiarity with the methodology, which, together with their complementary knowledge and backgrounds, enabled a more nuanced and in-depth analysis of the empirical material.

## Conclusion

This study, exploring how the deployment of three eHealth applications affects the work of healthcare professionals, particularly nurses, indicates that new digital work routines change the work of nurses. Work generate by eHealth applications involves data supervision, reassurance about reported and unreported data, and interpretation of patient-generated data, in addition to laying out/editing data into electronic health records. The eHealth applications influence caregiving and demands nurses’ attention. The extra nurturing required to take care of the eHealth applications, encourage patients to use them, and manage patient data becomes additional and invisible labour for nurses. As eHealth application usage continues to increase worldwide, steps are needed to ensure that the development does not create more invisible work for nurses. Thus, the use of eHealth applications in practice is not a technological fix that automatedly makes the work of nurses more efficient and easier—instead eHealth application changes the work of nurses by bringing new dimensions of the nursing–technology relationship into practice. This needs to be recognised and acknowledged.

## Data Availability

The datasets generated and/or analysed during the current study are not publicly available due to that the participants of this study did not give written consent for their data to be shared publicly. However, some of the data is available from the corresponding author (susanne.frennert@design.lth.se) on reasonable request and if permission from the Swedish Ethical Review Authority.

## References

[1] Eysenbach G (2001). What is e-health?. J Med Internet Res.

[2] van der Kleij RM, Kasteleyn MJ, Meijer E, Bonten TN, Houwink EJ (2019). SERIES: eHealth in primary care. Part 1: Concepts, conditions and challenges. Eur J Gen Pract.

[3] Boers SN, Jongsma KR, Lucivero F, Aardoom J, Büchner FL, de Vries M (2020). SERIES: eHealth in primary care. Part 2: exploring the ethical implications of its application in primary care practice. Eur J Gen Pract.

[4] Ossebaard HC, Van Gemert-Pijnen L (2016). eHealth and quality in health care: implementation time. Int J Qual Health Care.

[5] Hellberg S, Johansson P (2017). eHealth strategies and platforms–The issue of health equity in Sweden. Health Policy and Technology.

[6] Wernhart A, Gahbauer S, Haluza D (2019). eHealth and telemedicine: Practices and beliefs among healthcare professionals and medical students at a medical university. PLoS ONE.

[7] McBain H, Shipley M, Newman S (2015). The impact of self-monitoring in chronic illness on healthcare utilisation: a systematic review of reviews. BMC Health Serv Res.

[8] Koehler F, Koehler K, Deckwart O, Prescher S, Wegscheider K, Kirwan B-A (2018). Efficacy of telemedical interventional management in patients with heart failure (TIM-HF2): a randomised, controlled, parallel-group, unmasked trial. Lancet.

[9] Cajander Å, Moll J, Englund S, Hansman A. Medical Records Online for Patients and Effects on the Work Environment of Nurses. InMIE; 2018. p. 271–275. 29677965

[10] Öberg U, Orre CJ, Isaksson U, Schimmer R, Larsson H, Hörnsten Å (2018). Swedish primary healthcare nurses’ perceptions of using digital eH ealth services in support of patient self-management. Scand J Caring Sci.

[11] Goodare P (2017). Literature review: Why do we continue to lose our nurses?. Aust J Adv Nurs.

[12] Godinho MA, Martins H, Al-Shorbaji N, Quintana Y, Liaw S-T (2022). “Digital Health Diplomacy” in Global Digital Health? A call for critique and discourse. J Am Med Inform Assoc.

[13] Valokivi H, Carlo S, Kvist E, Outila M. Digital ageing in Europe: A comparative analysis of Italian, Finnish and Swedish national policies on eHealth. Ageing & Society. 2023;43(4):835–56.

[14] Daniels AK (2014). Invisible Work*. Soc Probl.

[15] Hatton E (2017). Mechanisms of invisibility: rethinking the concept of invisible work. Work Employ Soc.

[16] Allen D. The invisible work of nurses: hospitals, organisation and healthcare. Routledge; 2014.

[17] Henderson A (2001). Emotional labor and nursing: An under-appreciated aspect of caring work. Nurs Inq.

[18] Palmer E, Eveline J (2012). Sustaining low pay in aged care work. Gend Work Organ.

[19] Star SL, Strauss A (1999). Layers of silence, arenas of voice: The ecology of visible and invisible work. Comput supported cooperative work (CSCW).

[20] Hampson I, Junor A (2005). Invisible work, invisible skills: interactive customer service as articulation work. N Technol Work Employ.

[21] Oudshoorn N (2008). Diagnosis at a distance: the invisible work of patients and healthcare professionals in cardiac telemonitoring technology. Sociol Health Illn.

[22] Bødker M, Juul Nielsen A. Providing rehabilitation online–invisible work and diagnostic agents. J Health Organ Manag. 2015;29(7):948–64. 10.1108/JHOM-06-2014-0091 26556161

[23] Blouin AS, Podjasek K (2019). The continuing saga of nurse staffing: Historical and emerging challenges. J Nurs Adm.

[24] Punshon G, Maclaine K, Trevatt P, Radford M, Shanley O, Leary A (2019). Nursing pay by gender distribution in the UK-does the Glass Escalator still exist?. Int J Nurs Stud.

[25] Stockdale M, Warelow PJ (2000). Is the complexity of care a paradox?. J Adv Nurs.

[26] Elwér S, Aléx L, Hammarström A (2010). Health against the odds: Experiences of employees in elder care from a gender perspective. Qual Health Res.

[27] Pietroni PC (1991). Stereotypes or archetypes? A study of perceptions amongst health-care students. J Soc Work Pract.

[28] Frechette J, Carnevale FA (2020). Exploring a hermeneutic perspective of nursing through revisiting nursing health history. Nurs Philos.

[29] Wajcman J (2010). Feminist theories of technology. Camb J Econ.

[30] Ensmenger N, Misa TJ (2010). Making programming masculine. Gender codes: Why women are leaving computing.

[31] Barnard A. Philosophy of technology and nursing. Nurs Philos. 2002;3(1):15–26.

[32] Sandelowski M (1999). Troubling distinctions: a semiotics of the nursing/technology relationship. Nurs Inq.

[33] Barnard A (2002). Philosophy of technology and nursing. Nurs Philos.

[34] Cassell J (2004). Stories, moral judgment, and medical care in an intensive care unit. Qual Health Res.

[35] Hughes EC, Riesman D, Becker HS. The sociological eye: Selected papers. Routledge; 2017.

[36] Dillard-Wright J (2019). Electronic health record as a panopticon: A disciplinary apparatus in nursing practice. Nurs Philos.

[37] Akrich M, Bijker WE, Law J (1992). The de-scription of technical objects. Shaping technology/building society: Studies in sociotechnical change.

[38] Golz C, Peter KA, Müller TJ, Mutschler J, Zwakhalen SM, Hahn S (2021). Technostress and digital competence among health professionals in Swiss psychiatric hospitals: cross-sectional study. JMIR mental health.

[39] Nazeha N, Pavagadhi D, Kyaw BM, Car J, Jimenez G, Car LT (2020). A digitally competent health workforce: scoping review of educational frameworks. J Med Internet Res.

[40] De Leeuw JA, Woltjer H, Kool RB (2020). Identification of factors influencing the adoption of health information technology by nurses who are digitally lagging: in-depth interview study. J Med Internet Res.

[41] Ziebland S, Hyde E, Powell J (2021). Power, paradox and pessimism: on the unintended consequences of digital health technologies in primary care. Soc Sci Med.

[42] Walsham G (1995). Interpretive case studies in IS research: nature and method. Eur J Inf Syst.

[43] Flyvbjerg B (2006). Five misunderstandings about case-study research. Qual Inq.

[44] Yin RK (2009). How to do better case studies. The SAGE handbook of applied social research methods.

[45] Oudshoorn N (2009). Physical and digital proximity: emerging ways of health care in face-to-face and telemonitoring of heart-failure patients. Sociol Health Illn.

[46] Cajander Å, Larusdottir M, Hedström G (2020). The effects of automation of a patient-centric service in primary care on the work engagement and exhaustion of nurses. Quality and User Experience.

[47] Brooks J, Horrocks C, King N. Interviews in qualitative research. Interviews in qualitative research. 2018;1–360.

[48] Carpiano RM (2009). Come take a walk with me: The “Go-Along” interview as a novel method for studying the implications of place for health and well-being. Health Place.

[49] Elo S, Kyngäs H (2008). The qualitative content analysis process. J Adv Nurs.

[50] Frennert S, Petersson L, Muhic M, Rydenfält C, Nymberg VM, Ekman B (2022). Materiality and the mediating roles of eHealth: A qualitative study and comparison of three cases. Digital health.

[51] Walsham G (2006). Doing interpretive research. Eur J Inf Syst.

[52] Hsieh H-F, Shannon SE (2005). Three approaches to qualitative content analysis. Qual Health Res.

[53] Creswell JW, Poth CN. Qualitative inquiry and research design: Choosing among five approaches. Sage publications; 2016.

[54] Kraus S, Schiavone F, Pluzhnikova A, Invernizzi AC (2021). Digital transformation in healthcare: Analyzing the current state-of-research. J Bus Res.

[55] Massaro M. Digital transformation in the healthcare sector through blockchain technology. Insights from academic research and business developments. Technovation. 2021;7:102386.

[56] Nardi BA, Engeström Y (1999). A web on the wind: The structure of invisible work. Comput Support Coop Work.

[57] Suchman L (1995). Making work visible. Commun ACM.

[58] Suchman MC (1995). Managing legitimacy: Strategic and institutional approaches. Acad Manag Rev.

[59] Stroebaek PS (2013). Let's have a cup of coffee! Coffee and coping communities at work. Symb Interact.

[60] Lie SS, Karlsen B, Graue M, Oftedal B (2019). The influence of an eHealth intervention for adults with type 2 diabetes on the patient–nurse relationship: a qualitative study. Scand J Caring Sci.

[61] Schwennesen N (2021). Between repair and bricolage: digital entanglements and fragile connections in dementia care work in Denmark.

[62] Engesmo J, Panteli N (2020). Invisible Work Pre-and Post-Digitalisation. The case of a health authority in Norway. Scandinavian J Information Systems.

[63] Moreno-Llamas A, García-Mayor J, De la Cruz-Sánchez E (2020). The impact of digital technology development on sitting time across Europe. Technol Soc.

[64] Frennert S, Erlingsdóttir G, Muhic M, Rydenfält C, Milos Nymberg V, Ekman B. ‘It increases my ability to influence my ways of working’: A qualitative study on digitally mediated patient management in primary healthcare. Scand J Caring Sci. 2023;37(1):88–105. 10.1111/scs.13099 35833314

[65] Carboni C, Wehrens R, van der Veen R, de Bont A (2022). Conceptualizing the digitalization of healthcare work: A metaphor-based Critical Interpretive Synthesis. Soc Sci Med.

[66] Nicolini D. Practice theory, work, and organization: An introduction. OUP Oxford; 2012.

[67] Archibald MM, Ambagtsheer RC, Casey MG, Lawless M (2019). Using zoom videoconferencing for qualitative data collection: perceptions and experiences of researchers and participants. Int J Qual Methods.

